# A survey of nongovernmental organizations on their use of WHO’s prequalification programme

**DOI:** 10.2471/BLT.19.233882

**Published:** 2020-04-07

**Authors:** Ariadna Nebot Giralt, Maya Ronse, Raffaella Ravinetto

**Affiliations:** a QUAMED, Brussels, Belgium.; b Public Health Department, Institute of Tropical Medicine, Nationalestraat 155, 2000 Antwerp, Belgium.

## Abstract

**Objective:**

To obtain the perspectives of some small- and medium-sized organizations on the World Health Organization (WHO) prequalification programme for medicines and to ascertain organizations’ unmet needs.

**Methods:**

We conducted an exploratory, qualitative study in 2018 among 17 representatives of 15 small- and medium-sized Belgian and non-Belgian organizations who purchase medicines for humanitarian, development or public programmes in low- and middle-income countries. We used semi-structured interviews to obtain respondents’ views and experiences of using WHO prequalification guidance when procuring medicines. We identified emerging themes and formulated recommendations about the activities of the WHO Prequalification Team.

**Findings:**

Most respondents suggested expanding prequalification to essential antibiotics, particularly paediatric formulations; and insulin, antihypertensives and cancer treatments. Respondents were concerned about irregular availability of WHO-prequalified medicines in the marketplace and sometimes high prices of prequalified products. Small organizations, in particular, had difficulties negotiating low-volume purchases. Organizations working in primary health care and hospitals seldom referred to the prequalified lists.

**Conclusion:**

We recommend that the WHO-prequalified products be expanded to include essential antibiotics and medicines for noncommunicable diseases. The WHO Prequalification Team could require prequalified manufacturers to make publicly available the details of their authorized distributors and facilitate a process of harmonization of quality assurance policies across all donors. Prequalification of distributors and procurement agencies could help create more transparent and stringent mechanisms. We urge WHO Member States and funders to sustain support for the WHO Prequalification Team, which remains important for the fulfilment of universal health coverage.

## Résumé

**Objectif:**

Connaître le point de vue de quelques petites et moyennes organisations vis-à-vis du Programme de préqualification des médicaments dirigé par l'Organisation mondiale de la Santé (OMS), et déterminer quels sont les besoins non satisfaits de ces organisations.

**Méthodes:**

Nous avons mené une étude qualitative exploratoire en 2018 auprès de 17 représentants de 15 petites et moyennes organisations belges et non belges qui se procurent des médicaments pour des programmes humanitaires, publics ou de développement dans des pays à faible et moyen revenu. Nous avons conduit des entretiens semi-structurés afin de recueillir les avis et expériences des répondants concernant les conseils de préqualification fournis par l'OMS au moment d'acheter des médicaments. Nous avons identifié de nouveaux thèmes et formulé des recommandations autour des activités de l'équipe de préqualification de l'OMS**.**

**Résultats:**

La plupart des répondants ont suggéré d'élargir la préqualification aux antibiotiques essentiels, en particulier les formulations pédiatriques, ainsi qu'à l'insuline, aux antihypertenseurs et aux traitements contre le cancer. Plusieurs répondants ont exprimé leur inquiétude quant à la disponibilité irrégulière des médicaments préqualifiés par l'OMS sur le marché, mais aussi aux prix parfois élevés des produits préqualifiés. De leur côté, les petites organisations éprouvent des difficultés à négocier l'achat de faibles volumes. Enfin, les organisations qui travaillent dans le secteur des hôpitaux et soins de santé primaires se réfèrent rarement aux listes de préqualification.

**Conclusion:**

Nous recommandons de compléter la liste des produits préqualifiés établie par l'OMS afin d'inclure des médicaments et antibiotiques essentiels pour les maladies non transmissibles. Par ailleurs, l'équipe de préqualification de l'OMS pourrait exiger des fabricants préqualifiés de publier les coordonnées de leurs distributeurs agréés et de favoriser l'harmonisation des politiques d'assurance de la qualité chez tous leurs donateurs. La préqualification des distributeurs et des organismes d'achat pourrait contribuer à créer des mécanismes plus rigoureux et transparents. Nous encourageons les bailleurs de fonds et États Membres de l'OMS à soutenir durablement l'équipe de préqualification de l'OMS, qui demeure un outil important dans l'instauration d'une couverture maladie universelle.

## Resumen

**Objetivo:**

Obtener las perspectivas de algunas organizaciones pequeñas y medianas sobre el programa de precalificación de medicamentos de la Organización Mundial de la Salud (OMS) y determinar las necesidades que las organizaciones no han cubierto.

**Métodos:**

En 2018 se realizó un estudio cualitativo y exploratorio entre 17 representantes de 15 organizaciones pequeñas y medianas belgas y no belgas que compran medicamentos para programas humanitarios, de desarrollo o públicos en países de ingresos bajos y medios. Se utilizaron entrevistas semiestructuradas para conocer las opiniones y las experiencias de los encuestados sobre el uso de la orientación de precalificación de la OMS en la compra de los medicamentos. Se identificaron temas emergentes y se formularon recomendaciones sobre las actividades del Equipo de precalificación de la OMS.

**Resultados:**

La mayoría de los encuestados sugirieron que se ampliara la precalificación a los antibióticos esenciales, en particular a los medicamentos pediátricos, y a la insulina, los antihipertensivos y los tratamientos contra el cáncer. Los encuestados se mostraron preocupados por la disponibilidad irregular de los medicamentos precalificados por la OMS en el mercado y, a veces, por los precios altos de los productos precalificados. En particular, las organizaciones pequeñas tenían dificultades para negociar las adquisiciones de bajo volumen. Las organizaciones que trabajaban en la atención primaria de la salud y en los hospitales se remitían a las listas de medicamentos precalificados pocas veces.

**Conclusión:**

Se recomienda que los productos precalificados por la OMS se amplíen para incluir los antibióticos y los medicamentos esenciales de las enfermedades no transmisibles. El Equipo de precalificación de la OMS podría exigir a los fabricantes precalificados que publiquen los datos de sus distribuidores autorizados y faciliten un proceso de armonización de las políticas relacionadas con la garantía de calidad para todos los donantes. La precalificación de los distribuidores y de las agencias de adquisiciones podría ayudar a crear mecanismos más transparentes y rigurosos. Se insta a los Estados miembros de la OMS y a los financiadores a que mantengan su apoyo al Equipo de precalificación de la OMS, que sigue siendo importante para el cumplimiento de la cobertura sanitaria universal.

## ملخص​

الغرض​ الحصول على وجهات النظر الخاصة ببعض المنظمات الصغيرة والمتوسطة الحجم بشأن برنامج التأهيل المسبق لمنظمة الصحة العالمية (WHO) للأدوية، والتأكد من الاحتياجات التي لم يتم الوفاء بها للمنظمات.

الطريقة قمنا بإجراء دراسة استكشافية ونوعية في عام 2018 بين 17 ممثلاً عن 15 منظمة بلجيكية وغير بلجيكية صغيرة ومتوسطة الحجم، والتي قامت بشراء الأدوية للبرامج الإنسانية أو التنموية أو العامة في الدول المحدودة الدخل والمتوسطة الدخل. قمنا بالاستعانة بالمقابلات الشخصية شبه المنظمة للحصول عليها آراء وخبرات المشاركين حول استخدام إرشادات التأهيل المسبق الخاصة بمنظمة الصحة العالمية عند شراء الأدوية. وحددنا المواضيع الناشئة وقمنا بصياغة توصيات بخصوص أنشطة فريق التأهيل المسبق لمنظمة الصحة العالمية.

النتائج اقترح معظم المشاركين توسيع التأهيل المسبق ليشمل المضادات الحيوية الأساسية، وبخاصة التركيبات الخاصة بالأطفال؛ والإنسولين، ومخفضات ضغط الدم المرتفع، وعلاجات السرطان. وأعرب المشاركون عن قلقهم من عدم انتظام توافر أدوية التأهل المسبق للمنظمة الصحة العالمية في السوق، وأحيانًا الأسعار المرتفعة لمنتجات التأهل المسبق. واجهت المنظمات الصغيرة، على وجه الخصوص، صعوبات في التفاوض بخصوص عمليات الشراء منخفضة الحجم. ونادراً ما تم الإشارة في قوائم التأهل المسبق إلى المنظمات العاملة في الرعاية الصحية الأولية والمستشفيات.

الاستنتاج نحن نوصي بتوسيع منتجات التأهيل المسبق الخاصة بمنظمة الصحة العالمية، لتشمل المضادات الحيوية الأساسية وأدوية الأمراض غير المعدية. يمكن أن يطلب فريق التأهيل المسبق، التابع لمنظمة الصحة العالمية، من المصنعين المؤهلين مسبقاً أن يوفروا تفاصيل الموزعين المعتمدين لديهم بشكل علني، وتسهيل عملية تنسيق سياسات ضمان الجودة لدى كل الجهات المانحة. يمكن للتأهيل المسبق أن يساعد الموزعين ووكالات المشتريات في وضع آليات أكثر شفافية وصرامة. ونحن نحث الدول الأعضاء وجهات التمويل في منظمة الصحة العالمية، على مواصلة الدعم لفريق التأهيل المسبق بمنظمة الصحة العالمية، والذي لا يزال مهماً لتحقيق التغطية الصحية الشاملة.

## 摘要

**目的:**

旨在了解一些中小型组织对世界卫生组织 (WHO) 药品资格预审项目的看法，并确定这些组织未满足的需求。

**方法:**

我们于 2018 年对 15 个中小型比利时和非比利时组织中的 17 名代表进行了探索性定性研究，这些代表购买的药物用于中低收入国家的人道主义、发展或公共项目。我们采用半结构式访谈的方式，获取了受访者对采购药品时使用世卫组织资格预审指南的看法和体验。我们确定了新主题，并对世卫组织资格预审小组的活动提出了建议。

**结果:**

大多数受访者建议扩大基本抗生素的资格预审范围，尤其是儿科制剂；以及胰岛素、降压药和癌症治疗药物。受访者担心世卫组织预审药品在市场上供应不稳定，有时预审产品的价格过高。特别是小型组织在商洽小批量采购时遭遇困难。初级医疗护理机构和医院的下属组织很少参考预审清单。

**结论:**

我们建议将世卫组织资格预审的药物扩大至包括基本抗生素和治疗非传染性疾病的药物。世卫组织资格预审小组可要求通过资格预审的制造商公开其授权经销商的详细信息，并帮助制定协调各捐助方之间质量保证政策的流程。经销商和采购机构的资格预审有助于建立更加透明和严格的机制。我们敦促世卫组织成员国和资助者继续支持世卫组织资格预审小组，这对实现全民健康覆盖意义非凡。

## Резюме

**Цель:**

Получить мнение некоторых малых и средних организаций по программе Всемирной организации здравоохранения по предварительной квалификации лекарственных средств и выяснить неудовлетворенные потребности организаций.

**Методы:**

В 2018 году авторы провели экспериментальное качественное исследование с участием 17 представителей от 15 организаций малого и среднего бизнеса в Бельгии и за ее пределами, которые закупают лекарственные средства для гуманитарных программ, программ по развитию или общественных программ, проводимых в странах с низким и средним уровнем дохода. Авторы использовали полуструктурированные интервью, чтобы узнать мнения и опыт респондентов в отношении использования руководства ВОЗ по предварительной квалификации при закупке лекарственных средств. Были определены новые темы, а также сформулированы рекомендации для деятельности группы предварительной квалификации ВОЗ.

**Результаты:**

Большинство респондентов предложили распространить действие предварительной квалификации на основные антибиотики, особенно на педиатрические препараты, а также инсулин, гипотензивные средства и медицинские препараты для лечения рака. Респонденты были обеспокоены нерегулярным наличием лекарственных средств, предварительно квалифицированных ВОЗ, на рынке и высокими ценами на предварительно квалифицированные товары. В частности, малые организации сталкивались с трудностями при проведении переговоров о закупках в небольших объемах. Организации, работающие в учреждениях первичной медико-санитарной помощи и больницах, редко упоминались в списках предварительной квалификации.

**Вывод:**

Авторы рекомендуют расширить список товаров, предварительно квалифицированных ВОЗ, включив в них основные антибиотики и лекарственные средства для лечения неинфекционных заболеваний. Группа предварительной квалификации ВОЗ может потребовать от предквалифицированных производителей публиковать подробную информацию об уполномоченных дистрибьюторах и содействовать процессу согласования политики обеспечения качества со всеми финансирующими организациями. Предварительная квалификация дистрибьюторов и закупочных организаций поможет создать более прозрачные и строгие механизмы контроля и управления. Авторы призывают государства-члены ВОЗ и спонсоров оказывать поддержку группе предварительной квалификации ВОЗ, которая важна для обеспечения всеобщего охвата медико-санитарными услугами.

## Introduction

A key element of achieving universal health coverage in target 3.8 of the sustainable development goals is “access to safe, effective, quality and affordable essential medicines and vaccines for all.” Ensuring the quality of medicines is a moral imperative for any concerned stakeholder,^1^ and failure to do so may have major public health and economic consequences. The globalization of production and distribution, however, coupled with the weakness of national medicines regulatory authorities in many low- and middle-income countries, makes it difficult to thoroughly assess the quality of medicines available in the global market.^2^ Most national medicines regulatory authorities in low- and middle-income countries lack the human and financial resources and infrastructure to assess the efficacy, safety and quality of medical products submitted for marketing authorization or to maintain adequate postmarketing surveillance. The World Health Organization (WHO) has developed a global benchmarking tool for systematically evaluating regulatory systems.^3^ Yet, as reported in 2018, only 26% (50) of 194 WHO Member States have the capacity to enforce adequate quality assurance systems.^4^ Consequently, low- and middle-income countries are particularly at-risk of having poor-quality medicines that weaken their health systems and erode trust.^5^^–^^10^ Based on a literature review of papers that reported on field studies or surveys of the quality of medicines, WHO has estimated that about 10.5% of medicines in low- and middle-income countries are substandard or falsified (that is, 1166 of 11 156 tested samples in low-income countries and 3906 of 35 884 samples in middle-income countries).^11^

The WHO Pre-Qualification of Medicines Programme^12^ was launched in 2001 for guiding United Nations agencies with respect to the quality of medicines for treatment of human immunodeficiency virus infection and acquired immune deficiency syndrome (HIV/AIDS), tuberculosis and malaria. In 2013, the programme was merged with the WHO Pre-Qualification of Diagnostics Programme and Vaccines, to create the WHO Prequalification Team. The team provides a variety of services, including: assessment of quality control laboratories; training, advice and clarifications within the framework of its guidelines for national medicines regulatory authorities; and assessment of active pharmaceutical ingredients and finished pharmaceutical products for HIV/AIDS, malaria, tuberculosis, hepatitis, diarrhoea, influenza, reproductive health and neglected tropical diseases. The team’s role in assessing medicines consists of an in-depth, transparent assessment of all technical files of the product and inspection of manufacturing sites and contract research organizations.^13^ After awarding prequalification, the team ensures the ongoing quality of prequalified products by monitoring variations, periodical re-qualifications, reinspection of manufacturing sites and field quality surveys. WHO’s prequalification programme has raised awareness of the importance of quality assurance: a system by which quality is built into a product at every step of development, production and distribution.^1^^,^^3^

The findings of structured medicines quality surveys^14^^–^^16^ have demonstrated that WHO prequalification has had a major impact in assuring the quality of HIV/AIDS, malaria and tuberculosis medicines used in low- and middle-income countries and in reducing the likelihood that scarce resources are spent on products of unknown quality.^17^ The list of prequalified medicines by therapeutic area is available publicly^18^ and is an important tool for purchasers of medicines. As of 2014, WHO had prequalified 85–90% of antiretroviral, antimalarial and tuberculosis medicines, procured by or with funds from the Global Fund, United Nations Children’s Fund and Unitaid.^17^ The prequalified products lists are also used by nongovernmental organizations (NGOs) and other stakeholders, such as national procurement agencies. It is hoped that the positive experience with HIV/AIDS, malaria and tuberculosis medicines will be replicated in the other areas covered by the WHO Prequalification Team, such as hepatitis, diarrhoea, influenza, reproductive health and neglected tropical disease. Currently, in early 2020 however, relatively few essential medicines are covered by WHO prequalification. In addition, there are not yet prequalified products for some medicines in therapeutic areas under the team’s scope;^19^ for others, only one product has so far been prequalified.

Here, we aimed to gain an understanding of the perceptions of some small- and medium-sized organizations towards the WHO prequalification and to ascertain organizations’ unmet needs. We combined these responses with the reflections of our researchers to formulate recommendations on the activities of the WHO Prequalification Team.

## Methods

We conducted an exploratory, qualitative study between 10 September and 15 October 2018 among representatives and focal points of Belgian and non-Belgian organizations that are members of Be-Cause Health or QUAMED. Be-Cause Health is a Belgian network that facilitates exchange and capitalization of knowledge about international health and development cooperation, and hosts a medicines working group for members interested or involved in the management of medicines procured for and used in low- and middle-income countries.^20^ QUAMED is an independent not-for-profit organization that brings together NGOs and public or not-for-profit procurement centres, to improve access to quality medicines by raising awareness of and strengthening the quality assurance systems of its partners.^21^^,^^22^

We invited participation from all representatives of organizations that are members of the Be-Cause Health Medicines Working Group and procure medicines, and from all focal points of organizations that are members of QUAMED. All potential respondents already knew the researchers through their shared membership of the two platforms. We contacted participants by email, provided them with information about the study and, if they agreed to participate, made an appointment for an interview. The study was approved by the institutional review board of the Institute of Tropical Medicine, Antwerp, Belgium (ref. 1247/18). All respondents provided written consent for participation.

We collected the data by means of semi-structured interviews, to obtain an in-depth understanding of participants’ perspectives and perceptions of the WHO prequalification process and of the organization’s unmet needs. Interviews were conducted by telephone or in person, mostly in English and some in French or Spanish. The interview guide is available on our institutional website.^23^ The primary scope of the interviews was: to identify those essential medicines for which, to the best of the respondents’ and researchers’ knowledge, no quality-assured finished pharmaceutical products are currently available on the market; and to formulate recommendations about possible expansion of the activities of the WHO Prequalification Team. The interviewers first addressed the general challenges related to procurement and purchase of medicines for field medical programmes, and gradually moved to the core issues of the survey, that is, which medicines are not available as prequalified products.

Handwritten notes were taken. The researchers who conducted the interviews analysed the data manually from the notes, using thematic content analysis, which focused on examining themes or patterns of themes from information collected during the interviews. After the systematic data analysis, we identified and listed the main themes, discussed them among our research team and, when needed, cross-checked them with the WHO prequalified products list.^18^ We also analysed whether specific patterns emerged related to the types or categories of organizations or respondents.

## Results

### Participants

We contacted potential respondents in 22 organizations; 19 people agreed to participate and 17 participants from 15 organizations attended the appointment for an interview. Our final sample included 17 representatives of 14 small- and medium-sized medical NGOs and one African procurement centre. Most interviewees were pharmacists (10 participants), followed by medical doctors or nurses (four participants). Eight participants had worked in the organization for 4 years or less, and six participants for 10 or more years. The majority had previous relevant experience in procurement of medicines and all participants had some degree of responsibility for quality assurance of purchased medicines. All the organizations purchased medical products for programmes in low- and middle-income countries, with different scopes and target populations, ranging from well-established primary health-care or hospital programmes to vertical programmes and emergency interventions.

### Programme scope

Most respondents expressed a wish that the WHO Prequalification Team would provide guidance in more critical fields than are currently covered. Most respondents agreed on expanding prequalification to essential antibiotics, such as penicillin, particularly for paediatric formulations. Similarly, medicines for noncommunicable diseases were viewed as an important area. Insulin, antihypertensives and medicines for treating different forms of cancers were specifically mentioned. Few respondents (one to three per item) also mentioned that WHO prequalification should assess large-volume intravenous infusion solutions, such as glucose infusions, which are easy to manufacture, but are harmful if substandard (for example, if they do not comply with sterility specifications);^24^ disinfectants and antiseptics; various medical devices; and ribavirin, lidocaine, diazepam and opioid analgesics. A few respondents observed that their organization’s needs remained unmet even in therapeutic areas within the scope of the WHO prequalification system. For example, the WHO-prequalified list of medicines for neglected tropical diseases contained few finished pharmaceutical products; and there were at the time of the study no prequalified finished pharmaceutical products for certain medicines for multidrug-resistant tuberculosis.

### Procurement

While our main question revolved around essential medicines for which no quality-assured finished pharmaceutical products were yet available, the interviews uncovered some other issues indirectly linked to prequalification that are necessary if WHO-prequalified medicines are to reach all those in need. Many participants observed that the procurement of WHO-prequalified finished pharmaceutical products was not always straightforward. For instance, a low volume of purchase can make an organization unattractive for suppliers: its orders are less likely to be accepted or prioritized, and price negotiation is more difficult. Several respondents noted that WHO-prequalified finished pharmaceutical products were not always readily available on the international, regional or local markets. In addition, prices may vary considerably between suppliers. Some respondents, especially those in small organizations, expressed the view that WHO-prequalified products were more expensive than non-prequalified ones, limiting their capacity to procure quality-assured medicines. The difficulty was more pronounced in the case of dependency on a single supplier, when variability in pricing of WHO-prequalified finished pharmaceutical products was observed and when information about authorized distributors of prequalified products was not easily available. On a broader note, some representatives of small and medium-sized NGOs reminded us that they mainly purchased from wholesalers and distributors, rather than from manufacturers on a product-by-product basis. Participants observed that access to public, easy-to-access information about distributors of WHO-prequalified products, possibly by geographical region, and including transparent pricing information, would be helpful.

### Prequalification guidance

Our interviews revealed that knowledge and use of the WHO-prequalified lists of finished pharmaceutical products as practical tools for guiding procurement choices depended on the characteristics of a respondent’s organization. Only organizations that showed a strong institutional awareness of the need to assure pharmaceutical quality, and that had invested in quality assurance systems, seemed to be in a position to use the prequalified lists adequately and consistently. This includes for example, checking carefully the product and manufacturing site specifications.^1^ Organizations involved in vertical programmes in therapeutic areas covered by WHO prequalification, such as tuberculosis, were more likely to refer to the prequalified products lists on a regular basis. Small organizations, especially those working in primary health care and in hospitals, seldomly referred to the lists. Some respondents mentioned that they would benefit from a type of prequalification of distributors and procurement agencies, both internationally and in the regions and countries of intervention. Finally, the purchasing policies and practices of implementers that depended on external funding were strongly influenced by the funders’ quality assurance policy or lack of it.

## Discussion

Since we conducted this study, WHO has published an independent external review on the impact assessment of its prequalification and systems supporting activities. The review provides a perspective of the needs and interests of various stakeholders, including 16 donors or procurers.^25^ Nevertheless, we believe that the views revealed through our qualitative assessment are complementary and remain of interest for uncovering some specific views.

Respondents’ desire for expansion of prequalification to essential antibiotics and medicines for noncommunicable diseases is very relevant, given the contribution of poor-quality antibiotics to antimicrobial resistance^9^ and the epidemiological shift towards noncommunicable diseases in most low- and middle-income countries.^26^ It is noteworthy that in 2019 WHO started a pilot programme to prequalify human insulin. Another pilot programme is ongoing for two anticancer drugs and, in December 2019, the first biosimilar drug (trastuzumab) was prequalified. Respondents’ suggestions for other products outside the scope of the WHO prequalification system illustrate how concern about quality exists across the full range of essential medicines.^27^^–^^29^

Difficulties with negotiating procurement of WHO-prequalified medicines for small- and medium-sized NGOs echo the experiences of some central medical stores in Africa, which in a previous study reported poor transparency and lack of consideration from some suppliers.^21^ Our respondents’ concerns about the unavailability of WHO-prequalified finished pharmaceutical products in the market shows that organizations would benefit from access to more accessible information on suppliers or distributors of prequalified medicines, at regional or local level. It is notable that no respondents explicitly referred to the International Medical Products Price Guide^30^ as a tool to support negotiations, or to the Global Fund’s procurement tool, Wambo.^31^ This finding may indicate that these tools are not known to respondents or that the tools are not easy to use in respondents’ everyday professional life.

Our respondents’ comments about the cost of WHO-prequalified products highlights a possible drawback of quality assurance. Prequalified products may be more expensive than products that are not quality assured or not under the continuous regulatory oversight of a stringent quality assurance mechanism. On the one hand, higher cost can be justified by the harm reduction and the benefit to individual and public health. On the other hand, in many cases, manufacturers who invest in WHO-prequalified products are indirectly rewarded through access to bigger markets,^17^ providing them with an opportunity for economies of scale, resulting in lower production costs.^1^^,^^32^ This topic would benefit from additional research, to verify whether the prices of prequalified products are always significantly higher than those of non-prequalified competitors. If so, future research should assess whether this relates to the ex-factory price or to mark-ups along the distribution chain. Understanding whether prices could be better controlled would also be important, and, if so, how: via economies of scale, pooled negotiations and procurement, or enhanced transparency.

Our respondents from small organizations who were not involved in vertical programmes reported that they seldom referred to the WHO-prequalified products lists. The concept of prequalification is based on inspection of manufacturing sites and review of product dossiers; it results in the prequalification of a given finished pharmaceutical product from a given manufacturing site. Such an approach is relevant for big national and international purchasers, including some national procurement centres and major medical humanitarian NGOs. However, the approach seems to be less able to address the needs and capacities of purchasers in small- and medium-sized NGOs, and of implementers working in primary health care or hospitals with a broad range of essential medical products. These small or medium-sized organizations cannot individually address different manufacturers for purchasing on a product-by-product basis (that is, a given product by a given manufacturer), and they would benefit by a prequalification of distributors and suppliers.

We found that the quality assurance policies of funders affected organizations’ purchasing policies and practices. On a positive note, funders are increasingly becoming aware of the need for adequate quality assurance requirements in pharmaceutical procurement, especially when operating in countries where regulation of medicinal products is weak. For instance, some United Nations agencies, the United States Agency for International Development and the Bill and Melinda Gates Foundation are engaged in a process of harmonization of quality assurance policies.^33^ Among state funders, Belgium requires that implementers in low- and middle-income countries commit to ensuring the quality of procured medicines.^20^ However, when the funder does not prioritize quality assurance, and does not foresee a dedicated budget line, implementers are more likely to choose supply channels that are not fully secured, including medicines that are non-prequalified or not subject to stringent regulation. All funders should understand the relevance of pharmaceutical quality assurance; they should require adequate quality assurance criteria for the procurement of medicines in the programmes they fund; and they should make the guidance of the WHO Prequalification Team a mandatory requirement when WHO-prequalified products exist.^20^^,^^33^

Further investigation could look at whether the WHO Prequalification Team could facilitate or oversee a process of upgrade, when needed, and harmonization of quality assurance policies across all donors. This process could perhaps be done by building on the experience of the WHO’s Expert Review Panel, which provides guidance for time-limited procurement of much-needed products that are not yet prequalified or approved by a stringent regulatory authority.^1^

Our study had some limitations. We did not investigate how organizations make purchasing decisions in the absence of a WHO-prequalified product; thus, some possibly related issues did not emerge. For example, respondents did not mention whether they use the WHO’s Model Quality Assurance System for Procurement Agencies.^34^ While describing decision-making mechanisms in the absence of WHO prequalification guidance was not the main aim of this research, it would be a relevant subtopic for further research. Also, our findings drew on the views of a relatively small group of representatives of organizations that purchase medical products for public, humanitarian and development programmes for low- and middle-income countries. 

### Conclusions

We have formulated recommendations for how WHO and its Prequalification Team can enhance their capacity to meet procurers’ needs in the field. First, to address current trends in global health, invitations for expression of interest for WHO-prequalified products could be issued for essential antibiotics and medicines for noncommunicable diseases. Second, the WHO Prequalification Team could require manufacturers to make publicly available, in a dedicated website, for example, the lists, contacts and wholesaler prices of their authorized distributors, either acting internationally or regionally. Third, the WHO Prequalification Team could consider facilitating a process of harmonization of quality assurance policies across all donors.

Another recommendation emerged for WHO Member States and funders, given the importance of prequalification for protecting the health of the most vulnerable. In 2013, WHO began charging fees for activities related to medicines prequalification to improve the financial sustainability of the programme. We encourage stakeholders to sustain and increase support for the WHO prequalification programme, as a unique public good that is essential for the fulfilment of universal health coverage.

On another note, unrelated to the scope of the WHO Prequalification Team, we hope that transparent, stringent mechanisms can be put in place for a type of prequalification of wholesalers, distributors and procurement agencies. This could be helpful for small and medium-sized humanitarian, development and public-sector purchasers in low- and middle-income countries when seeking to procure quality-assured medical products.

## References

[1] Ravinetto R, Pinxten W, Rägo L. Quality of medicines in resource-limited settings: need for ethical guidance. Glob Bioet. 2018 9 18;29(1):81–94. 10.1080/11287462.2018.1522991 30245610 PMC6147095

[2] Caudron JM, Ford N, Henkens M, Macé C, Kiddle-Monroe R, Pinel J. Substandard medicines in resource-poor settings: a problem that can no longer be ignored. Trop Med Int Health. 2008 8;13(8):1062–72. 10.1111/j.1365-3156.2008.02106.x 18631318

[3] WHO global benchmarking tool (GBT) for evaluation of national regulatory system of medical products. National regulatory system (RS): indicators and fact sheets. Revision VI version 1. Geneva: World Health Organization; 2018. Available from: https://www.who.int/medicines/areas/regulation/01_GBT_RS_RevVI.pdf?ua=1 [cited 2019 Jun 17].

[4] Building regulatory capacity in countries to improve the regulation of health products. Geneva: World Health Organization; 2018. Available from: https://www.who.int/medicines/technical_briefing/tbs/TBS2018_RSS_Capacity_Building_CRSgroup.pdf?ua=1 [cited 2020 Mar 30].

[5] ’t Hoen E, Pascual F. Counterfeit medicines and substandard medicines: different problems requiring different solutions. J Public Health Policy. 2015 11;36(4):384–9. 10.1057/jphp.2015.22 26178809

[6] Newton PN, Amin AA, Bird C, Passmore P, Dukes G, Tomson G, et al. The primacy of public health considerations in defining poor quality medicines. PLoS Med. 2011 12;8(12):e1001139. 10.1371/journal.pmed.1001139 22162953 PMC3232210

[7] Johnston A, Holt DW. Substandard drugs: a potential crisis for public health. Br J Clin Pharmacol. 2014 8;78(2):218–43. 10.1111/bcp.12298 24286459 PMC4137817

[8] Newton PN, Caillet C, Guerin PJ. A link between poor quality antimalarials and malaria drug resistance? Expert Rev Anti Infect Ther. 2016 6;14(6):531–3. 10.1080/14787210.2016.1187560 27187060

[9] Nwokike J, Clark A, Nguyen PP. Medicines quality assurance to fight antimicrobial resistance. Bull World Health Organ. 2018 2 1;96(2):135–7. 10.2471/BLT.17.199562 29403117 PMC5791778

[10] Ravinetto R, Vandenbergh D, Macé C, Pouget C, Renchon B, Rigal J, et al. Fighting poor-quality medicines in low- and middle-income countries: the importance of advocacy and pedagogy. J Pharm Policy Pract. 2016 11 10;9(1):36. 10.1186/s40545-016-0088-0 27843547 PMC5105267

[11] Study on the public health and socioeconomic impact of substandard and falsified medical products. Geneva: World Health Organization; 2017. Available from: http://www.who.int/medicines/regulation/ssffc/publications/gsms-report-sf/en/[cited 2019 Mar 5].

[12] Essential medicines and health products: prequalification of medicines [internet]. Geneva: World Health Organization; c2020. Available from: https://extranet.who.int/prequal/ [cited 2020 Mar 24].

[13] Essential medicines and health products: prequalification of medicines. medicines/finished pharmaceutical products [internet]. Geneva: World Health Organization; 2020. Available from: https://extranet.who.int/prequal/content/prequalified-lists/medicines [cited 2019 Mar 5].

[14] Kuwana R, Sabartova J. Survey of the quality of selected antiretroviral medicines circulating in five African countries. WHO Drug Inf. 2017;31(2):162–5. Available from https://www.who.int/medicines/publications/druginformation/issues/WHO_DI_31-2_QualMonitoring.pdf [cited 2020 Mar 24].

[15] Survey of the quality of selected antimalarial medicines circulating in six countries of sub-Saharan Africa. Geneva: World Health Organization; 2011. Available from: https://www.who.int/medicines/publications/qamsareport/en/ [cited 2020 Mar 27].

[16] Survey of the quality of anti-tuberculosis medicines circulating in selected newly independent states of the former Soviet Union. Copenhagen: World Health Organization Regional Office for Europe; 2011. Available from: https://extranet.who.int/prequal/sites/default/files/documents/TBQuality-Survey_Nov2011_1.pdf [cited 2020 Mar 27].

[17] ’t Hoen EF, Hogerzeil HV, Quick JD, Sillo HB. A quiet revolution in global public health: the World Health Organization’s Prequalification of Medicines Programme. J Public Health Policy. 2014 5;35(2):137–61. 10.1057/jphp.2013.53 24430804

[18] Prequalified lists [internet]. Geneva: World Health Organization; c2020. Available from: https://extranet.who.int/prequal/content/prequalified-lists/medicines [cited 2020 Mar 24].

[19] Nurse-Findlay S, Taylor MM, Savage M, Mello MB, Saliyou S, Lavayen M, et al. Shortages of benzathine penicillin for prevention of mother-to-child transmission of syphilis: An evaluation from multi-country surveys and stakeholder interviews. PLoS Med. 2017 12 27;14(12):e1002473. 10.1371/journal.pmed.1002473 29281619 PMC5744908

[20] Ravinetto R, Roosen T, Dujardin C. The Belgian commitment to pharmaceutical quality: a model policy to improve quality assurance of medicines available through humanitarian and development programs. J Pharm Policy Pract. 2018 4 19;11(12):12. 10.1186/s40545-018-0136-z 29713474 PMC5907286

[21] Nebot Giralt A, Schiavetti B, Meessen B, Pouget C, Caudron JM, Marchal B, et al. Quality assurance of medicines supplied to low-income and middle-income countries: poor products in shiny boxes? BMJ Glob Health. 2017 3 29;2(2):e000172. 10.1136/bmjgh-2016-000172 28589013 PMC5435257

[22] Van Assche K, Nebot Giralt A, Caudron JM, Schiavetti B, Pouget C, Tsoumanis A, et al. Pharmaceutical quality assurance of local private distributors: a secondary analysis in 13 low-income and middle-income countries. BMJ Glob Health. 2018 6 9;3(3):e000771. 10.1136/bmjgh-2018-000771 29915671 PMC6001909

[23] Ravinetto R, Ronse M, Nebot Giralt A. The lack of quality-assured sources of medicines on the global market: a survey to explore the priority needs of purchasers in the Belgian humanitarian sector. Antwerp: Antwerp Institute Tropical Medicine; 2019. Available from: https://www.itg.be/Files/docs/190213WHOPQsurveyreport.pdf [cited 2019 Mar 20].

[24] Rauf A, Erum A, Noreen S, Shujaat J, Ashraf MU, Afreen S. Microbiological quality control of some non-sterile preparations commonly used in Pakistan. Pak J Pharm Sci. 2018 7;31(4):1237–42. 30033406

[25] Impact assessment of World Health Organization prequalification and systems supporting activities. Report of an Independent external review. Geneva: World Health Organization; 2019. Available from: https://www.who.int/medicines/Impact_assessment_WHO-PQ-RegSystems.pdf [cited 2019 Jun 17].

[26] Piot P, Caldwell A, Lamptey P, Nyrirenda M, Mehra S, Cahill K, et al. Addressing the growing burden of non-communicable disease by leveraging lessons from infectious disease management. J Glob Health. 2016 6;6(1):010304. 10.7189/jogh.06.010304 26955469 PMC4766788

[27] Peyraud N, Rafael F, Parker LA, Quere M, Alcoba G, Korff C, et al. An epidemic of dystonic reactions in central Africa. Lancet Glob Health. 2017 2;5(2):e137–8. 10.1016/S2214-109X(16)30287-X 28104176

[28] Mumphansha H, Nickerson JW, Attaran A, Overton S, Curtis S, Mayer P, et al. An analysis of substandard propofol detected in use in Zambian anesthesia. Anesth Analg. 2017 8;125(2):616–9. 10.1213/ANE.0000000000002226 28682949

[29] Ndichu ET, Ohiri K, Sekoni O, Makinde O, Schulman K. Evaluating the quality of antihypertensive drugs in Lagos State, Nigeria. PLoS One. 2019 2 13;14(2):e0211567. 10.1371/journal.pone.0211567 30759124 PMC6373917

[30] International medical products price guide [internet]. Medford: Management Sciences for Health; c2020. Available from: http://mshpriceguide.org/en/home/ [cited 2020 Mar 24].

[31] Procurement tools [internet]. Geneva: Global Fund to Fight AIDS, Tuberculosis and Malaria; c2020. Available from: https://www.theglobalfund.org/en/sourcing-management/wambo/ [cited 2020 Mar 24].

[32] Ravinetto R, Dujardin C. Universal health coverage: drug quality and affordability can go together. BMJ. 2019 10 15;367:l6004. 10.1136/bmj.l6004 31615789

[33] Guiding principles for donors regarding quality assurance of essential medicines and other health care commodities. Arlington: Partnership for Supply Chain Management; 2018. Available from: http://supplylines.pfscm.org/wp-content/uploads/2019/04/QA-Guiding-Principles-Final-2018.pdf [cited 2019 Jun 27].

[34] Model quality assurance system for procurement agencies. In: Annex III of the WHO Technical Report Series 986: WHO Expert Committee on Specifications for Pharmaceutical Preparations, forty-eighth report. Geneva: World Health Organization; 2014.

